# Loss-framing of information and pre-vaccination consultation improve COVID-19 vaccine acceptance: A survey experiment

**DOI:** 10.3389/fpubh.2023.1063444

**Published:** 2023-01-24

**Authors:** Kailu Wang, Eliza Lai-Yi Wong, Annie Wai-Ling Cheung, Dong Dong, Eng-Kiong Yeoh

**Affiliations:** ^1^Centre for Health Systems and Policy Research, The Jockey Club School of Public Health and Primary Care, Faculty of Medicine, The Chinese University of Hong Kong, Shatin, Hong Kong SAR, China; ^2^The Jockey Club School of Public Health and Primary Care, Faculty of Medicine, The Chinese University of Hong Kong, Shatin, Hong Kong SAR, China

**Keywords:** vaccine hesitancy, message framing, prospect theory, behavioral intervention, physician consultation, cash incentive

## Abstract

**Backgrounds:**

Vaccination remains one of the most effective ways to protect populations from COVID-19 infection, severe conditions, and death. This study aims to examine whether the gain/loss-framing of information, provision of subsidized pre-vaccination physician consultation, and cash incentives can improve COVID-19 acceptance amongst adults.

**Methods:**

A survey experiment was conducted within a broader cross-sectional survey of people aged 18–64 years in Hong Kong, China. The participants were randomly assigned to one of the eight groups derived from full-factorial design of the three strategies with stratification by age and sex. The vaccine acceptance rate was compared between people with and without any of the strategies. The heterogeneous effects of these strategies were identified for those with different perceptions of the pandemics and vaccine in multiple logistic regressions.

**Results:**

The survey experiment collected 1,000 valid responses. It found that loss-framed information and provision of subsidized physician consultation to assess suitability to be vaccinated, can improve vaccine acceptance, while cash incentives did not make a difference. The improvement effect of loss-framing information and physician consultation is stronger among those with higher perceived infection risk and severity of condition, as well as unvaccinated people with lower confidence in vaccine safety.

**Conclusions:**

The findings indicated that individualized loss-framing messages and equitable provision of subsidized pre-vaccination physician consultations can be incorporated in efforts to promote vaccine acceptance and vaccination roll-out speed. However, it remains inconclusive whether and how universal cash incentives may be deployed to support vaccination promotion.

## 1. Introduction

Vaccination remains one of the most important and effective ways to protect population from COVID-19 infection, severe conditions, and death (1–3). With the prevalence of multiple variants of SARS-CoV-2, it is likely that people would benefit from a third dose or annual booster of the vaccines to maintain an adequate level of protection, in addition to the standard two-dose schedule for most vaccines (4–6). However, vaccine hesitancy had reduced vaccination coverage and its roll-out speed, negatively impacting the capacity to slow and contain the spread of COVID-19 (7, 8). The growth in the proportion of people receiving at least one dose of vaccine slowed when vaccine coverage reached 60–70% in Hong Kong at the end of 2021 when this study was conducted (9)—while it has been estimated that coverage needs to reach 70–90% to achieve herd immunity (10). Therefore, more evidence-based policy options and communication strategies should be devised to improve vaccine acceptance in Hong Kong.

In the literature, there have been a large number of conjoint analysis, a method to elicit respondent's preference and perceived relative importance of different features or attributes of a product or service, identifying the vaccine characteristics influencing vaccine acceptance (11–17). Vaccine efficacy, safety and place of origin were found to be the most important characteristics that affect people's preference. However, these factors are difficult to modify when vaccines are already available. Regarding the modifiable factors, existing studies suggest that the importance of nearby vaccination locations and exemption of quarantine or social distancing measures for vaccinated people, may improve vaccine acceptance (18, 19). A recent study also found ownership-framing of the information in reminder, which induced psychological ownership of the vaccine by indicating the vaccine is “made available for you” that encourage people to “claim” their dose, may also improve vaccine uptake (20). Nevertheless, there are still various policy and intervention options to be considered for vaccination promotion, while there was limited evidence for them. In systematic reviews, it was summarized that there are different types of interventions for promoting COVID-19 vaccines, including communication (education/persuasion/psychological enablement), modeling (providing examples for imitation), environmental restructuring (changing context for vaccination), financial incentivization, restriction, and sanction (punishment) (21–23). The influence of modeling (influence of acquaintances), environmental restructuring (convenience of vaccination venues) and restrictions (different quarantine measures for vaccinated and unvaccinated people) were examined in a prior discrete choice study in Hong Kong (19), while the other factors were not tested in the local context. Among them, sanction or punishment for unvaccinated people were considered less acceptable by the public, and likely to cause social and psychological side effects and increase inequalities in health (22), so this was not considered in our study. Strategies related to communications and financial incentivization were, however, further tested in this study. Among communication strategies, healthcare professional consultation or recommendation serves as a way to improve vaccination through persuasion, assessment of safety of vaccination and potential risk of infection for vaccination, and information framing is a typical psychological enablement method in promotion of vaccination and other health prevention measures (22, 24). On the other hand, provision of cash for vaccinated individuals is a common way to financially incentivize people to receive the vaccine (23).

Therefore, this study aims to fill in the gaps by testing the influence of three strategies on vaccine acceptance, namely: gain/loss-framing of information (communication—psychological enablement); pre-vaccination physician consultation (communication—education/persuasion), and cash incentives (financial incentivization). First, information framing serves as a way to form or change people's attitudes through peripheral cues (i.e., messages that are framed differently) rather than carefully analyzing the information or arguments presented to them (25, 26). The gain/loss-framing is derived from prospect theory, which states that people are inclined to loss-aversion and more sensitive to losses than the gains with the same quantity (27). Gain-framing or loss-framing information would emphasize either the benefit (gain) or the cost (loss) of certain choices in the messages, respectively (28). Loss-framing was found effective in prevention behaviors with risks (e.g., side effect of vaccine) for health conditions with high susceptibility among certain groups of individuals (29–31). Therefore, while the effect of gain/loss-framing was considered inconclusive for vaccines against influenza, HPV, MMR, and hepatitis (32–36), it may nevertheless be effective for increasing COVID-19 vaccine acceptance, because of a relatively high perceived infection risk and lower confidence in its vaccines than in other vaccines, due to its rapid development and spread among populations (37). Second, as doctor's recommendation to vaccination is associated with higher vaccine acceptance (19), it is hypothesized that pre-vaccination physician consultations that assess whether the person is suitable to be vaccinated (i.e., the risk to have severe adverse events) may improve acceptance rate. Third, provision of cash incentives is considered as a common way to boost COVID-19 vaccine uptake in many countries (18, 38); however, they were uncommon for previous vaccination campaigns (39). The effect of this strategy is inconclusive in the local context, as an outcome inconsistent with overseas evidence was found (40). Therefore, it has been incorporated into this study as well. Moreover, to take into account individual psychosocial factors that may affect vaccine acceptance, the health belief model (HBM) was adopted. HBM states that an individual's course of action depends on the perceived susceptibility, perceived severity, benefit, barriers, and self-efficacy, as well as cues to action (19, 41, 42). It was also used to explain COVID-19 vaccination behaviors in a number of previous studies (43). Among these constructs, self-efficacy is rarely adopted in COVID-19 vaccination studies as successfully being vaccinated when the individual intends to do so usually does not require additional confidence in performing the behavior itself (43). For this study, the three strategies tested are: motivations for people to receive the vaccines (incentive to behave), which are considered to be cues to action under HBM; while other perceptions on COVID-19 infection and vaccination are measured for each respondent and controlled as covariates in the analysis.

## 2. Methods

### 2.1. Study sample and data collection

A survey experiment incorporated within an online cross-sectional survey was conducted among working-age adults aged 18–64 years in Hong Kong, China between 13 September 2021 and 8 November 2021. Slightly over half of the eligible population (around 57%) were fully vaccinated (two doses) at the beginning of the survey (44). Chinese adults aged between 18 and 64 years in Hong Kong were considered as target population of this survey. Those not living in Hong Kong at the moment of survey were excluded.

The roll-out speed of vaccination is as important as the eventual vaccination coverage of the population in an evolving situation of epidemics to prevent the disease spreading and reducing the number of people infected, hospitalized and dead. As vaccine uptake in Hong Kong population slowed before achieving potential herd immunity, we wish to find out whether relevant interventions can be applied to accelerate vaccination and increase the speed to achieve herd immunity. Therefore, apart from unvaccinated people, vaccinated people were also recruited to examine if the strategies could increase the roll-out speed by asking whether they would accept the vaccine earlier than they actually did. The participants were invited to take part through a participant panel with a well-stratified sample of working-age people, that were established in previous studies (19). Those who gave their consent to participate in future surveys on COVID-19 related topics in the panel, were approached and briefed for the study and its eligibility criteria through text message, followed by a link to the self-administered survey. The socio-demographical distribution of the sample were assessed during recruitment to adjust the target individuals to be invited based on the information provided at previous surveys. They were asked to give formal consent at the beginning of the survey, and then answer the screening questions for their eligibility (including their age and whether living in Hong Kong). The language used for the survey was Chinese. This study was reviewed and approved by the Survey and Behavioral Research Ethics Committee of the Chinese University of Hong Kong (SBRE-20-540). Informed consent was obtained from participants before the survey.

### 2.2. Design of survey experiment

For the survey experiment, three information blocks were designed as background information for the participants before indicating their vaccination acceptance (Table 1). The first information block is gain/loss-framing of information on vaccine effectiveness for reducing risk of COVID-19 infection and mortality (gain-framing vs. loss-framing)—which were designed based on gain/loss framed information for promoting vaccination and use of other medical products reported in previous studies (30, 45). The second information block involved showing how provision of subsidized physician consultation influenced the participants' suitability to be vaccinated, while the control group would not see this message (consultation vs. no consultation). The third information block involved showing provision of HK$500 (US$64) cash for fully vaccinated people as an incentive, while the control group would not see this message (cash vs. no cash). The amount of incentive was approximated with the 50-euro incentives used in a previous study (18).

**Table 1:** Messages on the three strategies used in the survey experiment.

**Information blocks**		**Messages shown in the questionnaire**
Information framing	Gain-framing	If you are vaccinated, you have around 1/2–1/20 probability of COVID-19 infection of un-vaccinated people, and have around 1/7–1/30 risk of death of un-vaccinated people.
	Loss-framing	If you are not vaccinated, you have around 2–20 times probability of COVID-19 infection of vaccinated people, and have around 7–30 times risk of death of vaccinated people.
Subsidized physician consultation	Not provided	Blank
	Provided	Government provides one fully subsidized physical examination and consultation by a doctor of your choice prior to vaccination to assess your suitability of vaccination, and the doctor tells you that you are suitable.
Cash incentive	Not provided	Blank
	Provided	The vaccinated individuals can receive HK$500 in cash.

Combining the three blocks, there were eight groups (2 × 2 × 2) of information that were different from each other in full-factorial design. The participants were first asked for their age, sex, and COVID-19 vaccination status, and then randomly assigned to one of the eight groups, stratified by age and sex. The randomization was performed using the built-in “randomizer” function in the widely-used Qualtrics (Provo, UT, USA) online survey platform, where the respondents have equal chance to be assigned to each one of the eight groups in the following survey experiment. For those who received at least one dose of vaccine, they were asked “under this context, when you have not received COVID-19 vaccine, would you receive the COVID-19 vaccine earlier than you actual did? (Yes/No)” after being shown the three information blocks. For those who did not receive any COVID-19 vaccine, they were asked “under this context, would you receive the COVID-19 vaccine in recent days? (Yes/No)” after the three information blocks. Six public health professionals and 10 adult members of the general public, belonging to different ages and occupations identified from our participant panel, who have similar cultural backgrounds as our study sample, were invited to review an assessment of the information blocks and survey experiment to improve its face validity and ensure study participants understood the questions and how to respond to them.

### 2.3. Measurements

Apart from the survey experiment, several socio-demographical characteristics and psychosocial perceptions were measured in the questionnaire. These measurements were designed based on previous studies on vaccination and the HBM (19, 41, 42), including (1) perceptions on COVID-19 pandemic, including their perceived risk of COVID-19 infection (HBM—perceived susceptibility; four-point scale, “very unlikely,” “not so likely,” “likely,” “very likely”), perceived severity of the condition if infected (HBM—perceived severity; four-point scale, “completely not severe,” “not so severe,” “slightly severe,” “very severe”), and level of concern over being quarantined (HBM—perceived severity; four-point scale, “completely concerned,” “not so concerned,” “concerned,” “very concerned”); (2) general perceptions on COVID-19 vaccines, including perceived safety (HBM—perceived barriers) and perceived effectiveness of the vaccine on oneself (HBM—perceived benefits; 10-point scale, point 6–10: relatively high perceived safety/effectiveness, point 1–5: relatively low perceived safety/effectiveness); and (3) socio-demographics, including age, sex, education level, household income, and whether having a chronic condition. The validity of these measurements was also reviewed by public health professionals and the members of public.

### 2.4. Statistical analysis

Sample characteristics were compared across different information groups to find out if there is any difference in their socio-demographical characteristics. The COVID-19 vaccine acceptance were compared between participants receiving different information as well as with different socio-demographical and psychosocial characteristics using cross-tabulation and chi-square test. Two multiple logistic regressions were applied for participants without (Model 1) and with (Model 2) COVID-19 vaccination records separately. The dependent variable was COVID-19 vaccine acceptance (or accept the vaccine earlier than actually did for those who received at least one dose of vaccine), and independent variables included three information blocks, namely gain/loss-framing on vaccine effectiveness, provision of subsidized physician consultation, and provision of cash incentives, and the participant characteristics including age, sex, whether having chronic conditions, education level, household income, perceived risk and perceived severity of COVID-19 infection, level of concerns over being quarantined, perceived effectiveness and perceived safety of COVID-19 vaccine. Vaccine acceptance rate was standardized for comparison across information groups according to age and sex distribution of the Hong Kong population aged 18–64 years. A direct standardization method was used to calculate the standardized acceptance rate by multiplying age- and sex-specific acceptance rate with population size of each of the age- and sex-groups in the reference population (i.e., Hong Kong population aged 18–64 years) (46). Following these two regressions, interactions between the three information blocks and perceived risk of COVID-19 infection (Model 3 and 4 for those who did not receive or received vaccines), perceived severity of condition (Model 5 and 6), perceived safety of the vaccines (Model 7 and 8), and perceived effectiveness of the vaccines (Model 9 and 10) were incorporated into different regressions individually, to find out whether or not the effects of the three information blocks would be modified by perceived susceptibility, severity, risk and benefit of COVID-19 and the vaccination.

## 3. Results

### 3.1. Sample characteristics

One thousand six hundred and fifteen invited adults agreed to be followed-up for participating in a COVID-19 related survey, while 1,299 of them actually agreed to participate in this survey. Amongst these, 299 did not complete or provide valid responses to the questionnaire, and the remaining 1,000 provided valid responses to the survey experiment and other key questions in this study. The response rate was 62.0%. Among them, there were more people under 35 years in the sample than the Hong Kong population because the participant panel was established based on previous online surveys and more younger people were included as they usually spend more time using the internet than middle and old-age adults (47), thus the sample was standardized with reference to population distributions during the analysis for the vaccine acceptance rate. The sample characteristics are shown in Supplementary Table 1. There were 56.2% with bachelor degree or above, 57.9% with HK$30,000 or above monthly household income, 12.9% with chronic conditions, and 80.7% received at least one dose of COVID-19 vaccine. Participants were randomly allocated to each of the eight information groups (group size ranged 124–126). No significant difference in these characteristics was found across these eight groups (all *P* > 0.05). In a previous study in Hong Kong, it was estimated that parental vaccine acceptance for their child aged 12–17 years were around 91% under loss-framed messages vs. 78% under gain-framed messages (19). The group difference is 13%. Therefore, using two-sided *Z*-test, it was estimated that each group/arm in the survey experiment should have 121 individuals to achieve 80% power to detect a difference between the group proportions of 13% at two-sided significance level of 0.05. We have at least 124 individuals in each subgroup, so the sample is sufficient to detect the difference.

### 3.2. Vaccine acceptance rate

**Figure 1:**
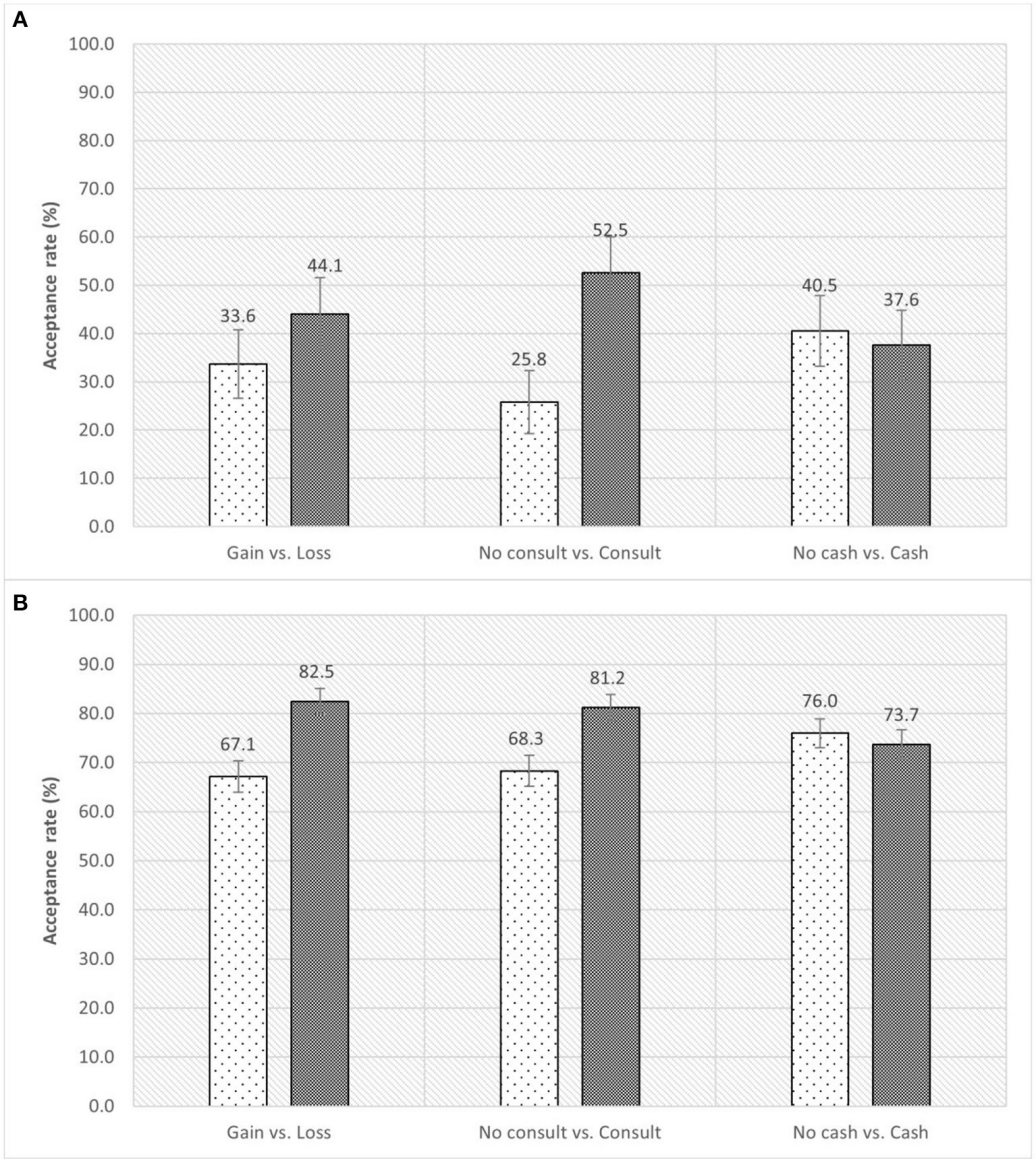
Vaccine acceptance rate for participants under different strategies. **(A)** Refers to the vaccine acceptance rate for those who did not receive COVID-19 vaccine. **(B)** Refers to the willingness to accept the vaccine earlier than actually did for those who received at least one dose of COVID-19 vaccine. The error bar refers to 95% confidence interval of the acceptance rate.

Figure 1 shows the comparison of COVID-19 vaccine acceptance of participants according to the information they received. Among those who did not receive the vaccine (*n* = 193), the standardized acceptance rate was significantly higher in the groups with provision of physician consultation for suitability to be vaccinated than those without the consultation [52.5% (95% confidence interval (CI): 45.1–60.0%) vs. 25.8% (95% CI: 19.2–32.3%), *P* < 0.001]. No differences were found between gain-framing and loss-framing of information [gain vs. loss: 33.6% (95% CI: 26.6–40.7%) vs. 44.1% (95% CI: 36.6–51.5%), *P* = 0.137], and between provisions of HK$500 cash incentives and no incentives [with vs. without cash: 37.6% (95% CI: 30.3–44.8%) vs. 40.5% (95% CI: 33.2–47.9%), *P* = 0.673]. Among those who received at least one dose of vaccine (*n* = 807), participants in the groups with loss-framing information [loss vs. gain: 82.5% (95% CI: 79.9–85.0%) vs. 67.1% (95% CI: 63.9–70.3%), *P* < 0.001] and physician consultation [with vs. without consultation: 81.2% (95% CI: 78.5–83.8%) vs. 68.2% (95% CI: 65.1–71.4%), *P* < 0.001] were more willing to receive the vaccine earlier than they did, while provision of cash incentives did not make a difference [with vs. without cash: 73.7% (95% CI: 70.7–76.7%) vs. 76.0% (95% CI: 73.0–78.9%), *P* = 0.433]. The vaccine acceptance across the socio-demographics and psychosocial perceptions can be found in Supplementary Tables 2, 3. Vaccine acceptance was also higher in people aged 35–49 years, without bachelor degree, perceived higher severity of conditions if infected, perceived higher safety and effectiveness of vaccines.

### 3.3. Effect of the strategies on vaccine acceptance

Table 2 shows the outcomes of multiple logistic regressions of vaccine acceptance for those who received and did not received vaccine separately (Model 1 and 2). For participants who did not receive the vaccine, consultation significantly improved the vaccine acceptance [adjusted odds ratio (AOR): 3.36, 95% CI: 1.57–7.18], while the effects of gain/loss-framing and cash incentives were not significant. This is similar to the findings in univariate analysis. Among the psychosocial perceptions to the pandemics and vaccines, those who had perceptions that the vaccine effectiveness is relatively high in general would be more likely to accept the vaccine in the future (AOR: 8.84, 95% CI: 2.34–33.45).

**Table 2:** Association between vaccine acceptance and the three strategies with adjustment of socio-demographic factors and psychosocial perceptions.

	**Not receive COVID-19 vaccine (Model 1**, ***n*** = **193)**		**Received at least one dose of vaccine (Model 2**, ***n*** = **807)**	
	**AOR** ^a^	**95% CI** ^a^	**AOR**	**95% CI**
Loss-framing (vs. gain-framing)	1.98	(0.92, 4.29)	2.33^**^	(1.64, 3.30)
Physician consultation (vs. no consultation)	3.36^*^	(1.57, 7.18)	2.12^**^	(1.50, 2.99)
Cash incentive (vs. no cash)	0.71	(0.33, 1.53)	0.91	(0.64, 1.28)
**Age (18–34 years as reference)**				
35–49 years	1.29	(0.50, 3.32)	1.31	(0.84, 2.05)
50–64 years	1.20	(0.47, 3.07)	0.82	(0.51, 1.31)
Female (vs. male)	0.78	(0.36, 1.71)	1.01	(0.71, 1.42)
With any chronic condition	0.57	(0.20, 1.59)	1.22	(0.73, 2.04)
Bachelor degree or above	0.45	(0.18, 1.10)	0.88	(0.59, 1.32)
HK$30,000+ monthly household income	0.92	(0.38, 2.19)	1.10	(0.74, 1.65)
Perceived “likely/very likely” to be infected	1.42	(0.60, 3.34)	1.25	(0.86, 1.82)
Perceived “slightly severe/very severe” if infected	1.98	(0.83, 4.70)	0.70	(0.48, 1.02)
Perceived relatively high vaccine safety	0.51	(0.12, 2.06)	2.26^**^	(1.45, 3.53)
Perceived relatively high vaccine effectiveness	8.84^*^	(2.34, 33.45)	1.96^*^	(1.24, 3.10)
“Slightly/very” concerned about being quarantined	0.85	(0.33, 2.14)	1.61^*^	(1.12, 2.31)

* P < 0.05.

** P < 0.001.

The regressions were applied to an age- and sex-standardized sample with reference to working-age population in Hong Kong.

a AOR, adjusted odds ratio; CI, confidence interval.

For participants who received the vaccine, provision of consultation (AOR: 2.33, 95% CI: 1.64–3.30) and loss-framing of information (AOR: 2.12, 95% CI: 1.50–2.99), could make them more willing to accept the vaccine earlier than they did. Cash incentives had little impact on vaccine acceptance. Besides these strategies, those who had confidence in the high level of safety (AOR: 2.26, 95% CI: 1.45–3.53) and effectiveness (AOR: 1.96, 95% CI: 1.24–3.10) of the vaccine, and higher level of concerns for being quarantined (AOR: 1.61, 95% CI: 1.12–2.31) were more likely to accept the vaccine earlier than they did.

### 3.4. Heterogeneous effects across different perceptions on the pandemics and vaccine

**Figure 2:**
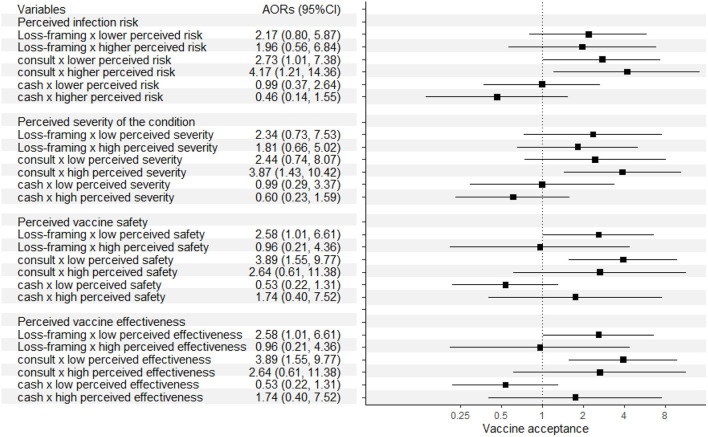
Effects of the three strategies according to different perceptions of the participants who did not received the vaccine.

Figure 2 shows the effects of the three strategies according to the different perceptions of the participants who did not receive the vaccine. Those with higher perceived risk of COVID-19 infection and higher perceived severity of the condition were more likely to be motivated to get vaccinated by subsidized physician consultation. Those who perceived themselves to have lower vaccination safety, were more likely to be influenced by loss-framing of information and physician consultation; while the effects of these strategies were similar between those with higher and lower perceived effectiveness of the vaccines.

**Figure 3:**
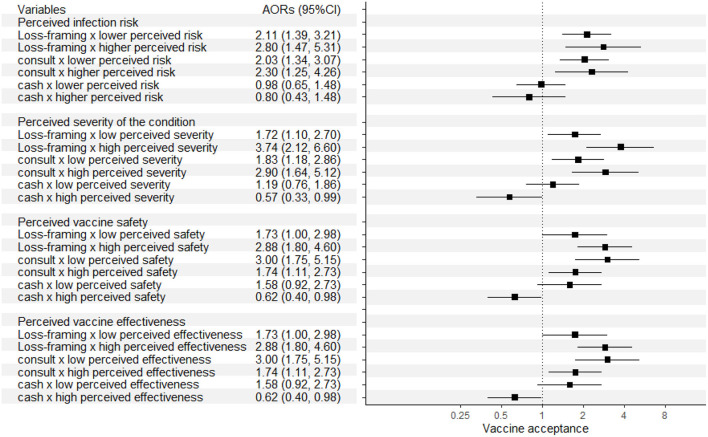
Effects of the three strategies according to different perceptions of the participants who received at least one dose of vaccine.

Figure 3 shows the effects of the strategies according to perceptions of participants who sreceived the vaccine. Those with higher perceived risk of sCOVID-19 infection and higher perceived severity of the condition, especially the latter, swere more likely to be influenced by loss-framing of information and physician consultation in willingness to get vaccinated earlier than they actually did. Similar to participants who did not receive the vaccine, those who perceived they had a lower vaccination safety were more likely to be influenced by physician consultation, but less likely to be influenced by loss-framing. Besides these, those who perceived a higher effectiveness of the vaccines were more likely to be influenced by loss-framing of information and physician consultation. The detailed outcomes of Model 3–10 can be found in Supplementary Tables 4–11.

## 4. Discussion

This study was conducted when 57% of the eligible population in Hong Kong were fully vaccinated; however, the vaccination coverage was not enough to provide herd immunity to protect the entire population (10). This survey experiment examined a few options for promoting vaccination under the HBM framework where three strategies are considered as cues to action and other HBM constructs are measured. It revealed that loss-framing of information and provision of subsidized physician consultation for examining suitability to be vaccinated, can improve the vaccine acceptance rate and vaccine roll-out speed—while the effect of HK$500 (US$64) cash incentives was not found. The other HBM constructs were found to have interactions over the effects of these cues to actions, as the influences of the three strategies were different across people with different perceptions on the pandemics and the vaccines.

In the survey experiment, loss-framing of information on vaccine effectiveness in terms of reducing risk of infection and mortality was found to be effective for increasing the willingness of those who received the vaccine to receive the vaccine earlier, which could improve the roll-out speed of vaccination coverage. The vaccine acceptance of those who did not receive the vaccine was higher when receiving loss-framing information, but the difference was not significant. As people tend to be more sensitive to losses than to gains of the same magnitude (27), people may perceive a higher level of utility losses resulted from vaccine refusal (i.e., being infected or death) in loss-framing context than gain-framing, and it exceeds the level of potential losses of getting vaccinated (i.e., discomfort/conditions/risk of death caused by vaccine side effects), while the perceived losses of vaccine refusal in gain-framing context was not higher than potential losses of getting vaccination (45). This finding is similar to a study that indicated loss-framing information could improve vaccination intentions in China (30). Other constructs of HBM that show perceptions on COVID-19 and its vaccine also have impacts on the effect of information framing. The effects of loss-framing were associated with a level of perceived safety of the vaccines to participants themselves, which is considered to be a barrier for vaccination under HBM. For people who did not receive a COVID-19 vaccine, the effect of loss-framing is weak among those who perceived lower safety of the vaccine, which may be because the loss-framing is not strong enough to make people ignore the potential risk of vaccination when they perceived the level of safety is low. Nevertheless, it could incentivize those who needed reassurance of higher safety to accept the vaccine, and prompt an earlier vaccination for those who may have hesitancy before.

The provision of a subsidized physician consultation could improve vaccine acceptance in both groups of people who received and did not receive the vaccine, which has rarely been tested as an intervention for promoting COVID-19 vaccination in previous research. Nevertheless, this finding is supported by several surveys that showed recommendation from doctors for vaccination play an important role in reducing vaccine hesitancy for vaccine against COVID-19 or other diseases (48–51). Another survey found that getting vaccinated at local doctors could also improve vaccine acceptance (18). Therefore, the influence of doctor consultation on vaccine acceptance, may be partially attributed to people's trust in their doctors (52). The consultation could also reduce people's concerns over vaccine safety and side effect, after the doctors have assessed patient's physical condition. In this survey, consulting a physician had a stronger impact on vaccine acceptance among those with lower perceived safety of vaccines than those with higher perceived safety, which supports the explanation that physician consultation could reduce people's concerns over vaccine safety. In other words, under HBM context, consultations can reduce the perceived barriers for participants getting vaccinated. This finding also implies that people who did not receive any vaccine dose until now, mainly do so out of concern over vaccine safety issue, which is consistent with previous local surveys that found safety concerns became increasing important in causing vaccine hesitancy (53).

The third options tested in the survey was provision of cash incentives (HK$500/US$64), which had no effect on COVID-19 vaccine acceptance. This finding is different from both the outcomes of studies in the US and European countries that revealed cash incentives (ranging from US$24 to over US$50) could improve vaccine acceptance by 2–4% (18, 38, 54), and the increment in vaccine uptake observed in Hong Kong after the announcement of a lottery draw of an apartment worth HK$10.8 million (55). However, another local study as well as a recent study in the US also found cash incentive does not work to increase vaccination uptake (39, 40). This may be caused by social desirability bias that people inclined not to admit they would accept the vaccine out of consideration of money in a hypothetical scenario where the cash incentive is not actually provided to the participants (56). Despite this, it is debatable whether cash incentives should be provided to promote vaccination. First, provision of incentives may be considered as a coercive method as it takes advantage of needs for money of people with lower socio-economic status, ignoring their autonomy and opinions over the vaccination (57, 58). Second, cash incentives may diminish the moral significance of getting vaccinated to protect oneself and others (58), which might jeopardize future vaccination promotion efforts as people may want to wait for incentives to get vaccinated. Third, a previous experiment indicated that high payment was associated with higher perceived risk for the task that the payer asked the study participant to do (59), which could also be why cash incentive was found to reduce vaccine acceptance for those who receive a vaccine and believe the vaccine's safety and effectiveness is high. It suggested that people would perceive vaccination as a risk if they are paid to do so, which is not helpful for promoting various vaccines.

Considering the findings, loss-framing of information and provision of subsidized physician consultation prior to vaccination could be helpful in promoting COVID-19 vaccination, particular for those with high perceived infection risk and severity of the condition, and unvaccinated people with concerns over vaccine safety. The findings on interactions between cues to actions and other HBM constructs indicate that it is important for clinicians and other public health professionals to understand individuals' perceptions on COVID-19 and the vaccine in practice, as it may influence the effect of different strategies for vaccination promotion. For use of loss-framing messages, individualized interventions should be considered in implementation rather than population-level dissemination of relevant messages, as loss-framing may induce powerless and fear in receivers, and cause anxiety among those who are more mentally vulnerable (60). Healthcare professionals can consider using the information framing strategies when an individual is deemed to be suitable for vaccine uptake and does not think the vaccine is unsafe, while this person may underestimate the benefits of vaccination (e.g., feeling there is no difference in being vaccinated or unvaccinated). Implementation of subsidized physician consultation can be first made available to older persons and people with pre-existing conditions, who are likely to have vaccine hesitancy and to have lower perceived safety toward the vaccines, while also possessing higher risk of experiencing critical conditions if infected (61). As for provision of physician consultation in the middle of a vaccination programme, there should be ways to reimburse those who have already been vaccinated before the subsidized consultation is launched to ensure an equal provision of publicly funded benefits.

This study possesses a few limitations. First, we recruited more people aged below 35 years and more females compared with the population in Hong Kong, which is why we adjusted for age and sex distribution in analysis for vaccine acceptance rate and its association with various factors. Second, we recruited both unvaccinated and vaccinated people to elicit their willingness to be vaccinated in the near future and earlier than they did in the past separately, so they were not combined for analysis because of the heterogeneous outcome measurements. By including vaccinated individuals, we aimed to find out whether relevant interventions can be used to accelerate their vaccination and increase vaccination roll-out speed—which is important in the evolving situation that characterizes epidemics. Meanwhile, the survey was conducted at a time when the third dose of vaccine was about to be made publicly available while there were no compulsory requirements or other incentives for people to accept the third dose. The acceptance rate of the vaccinated people can also be used to inform strategies to promote future booster dosage of COVID-19 vaccines. Nevertheless, the retrospective measurement of the latter group was not widely used in previous studies. There should be a larger sample of unvaccinated people recruited in future studies to find out the effect of these interventions amongst them.

## 5. Conclusion

This survey experiment found that loss-framing of information on vaccine effectiveness and provision of subsidized physician consultation prior to vaccination, could improve vaccine acceptance. Their effects are stronger for people with higher perceived infection risk and severity of condition, and unvaccinated people with low confidence over vaccine safety. The provision of universal cash incentives to encourage vaccination is a topic of debate, and its effect was inconclusive in our study's local context. Future studies could consider strategies to implement these interventions amongst the public, and examine their effectiveness in real-world contexts.

## Data availability statement

The datasets presented in this article are not readily available because of ethical reasons. Public availability of data would compromise confidentiality and privacy of participants. Requests to access the datasets should be directed to EW, lywong@cuhk.edu.hk.

## Ethics statement

The studies involving human participants were reviewed and approved by Survey and Behavioral Research Ethics Committee of the Chinese University of Hong Kong (SBRE-20-540). The patients/participants provided their written informed consent to participate in this study.

## Author contributions

KW conceptualized, designed, implemented the study, performed the data analysis, and drafted the manuscript. EW conceptualized, designed, and made critical revisions to the manuscript. AC contributed to acquisition of the data and data analysis. E-KY contributed to conceptualization of the study and made critical revisions to the manuscript. All authors edited and approved the final version of the manuscript.
