# Supplementary material for: Loss-framing of information and pre-vaccination consultation improve COVID-19 vaccine acceptance: A survey experiment
Source: Front Public Health. 2023 Jan 24;11:1063444. doi: 10.3389/fpubh.2023.1063444 (PMC9902910; doi:10.3389/fpubh.2023.1063444)
Supplement: Supplementary file 1 [file Table_1.DOCX]

Supplementary Material 1

**Supplementary tables and figures on subgroup characteristics and survey questionnaire**

Table S1. Sample characteristics according to eight groups with different messages.

|  | Gain | | Gain+consult | | Gain+cash | | Gain+consult+cash | | Loss | | Loss+consult | | Loss+cash | | Loss+consult+cash | | Total | | P value |
| --- | --- | --- | --- | --- | --- | --- | --- | --- | --- | --- | --- | --- | --- | --- | --- | --- | --- | --- | --- |
|  | N | % | N | % | N | % | N | % | N | % | N | % | N | % | N | % | N | % |  |
| **Age** |  |  |  |  |  |  |  |  |  |  |  |  |  |  |  |  |  |  |  |
| 18-34 yrs | 73 | 58.9 | 76 | 60.3 | 74 | 59.7 | 66 | 52.8 | 68 | 54.0 | 57 | 45.2 | 72 | 58.1 | 72 | 57.6 | 558 | 55.8 | 0.513 |
| 35-49 yrs | 29 | 23.4 | 29 | 23.0 | 30 | 24.2 | 42 | 33.6 | 35 | 27.8 | 42 | 33.3 | 35 | 28.2 | 35 | 28.0 | 277 | 27.7 |  |
| 50+ yrs | 22 | 17.7 | 21 | 16.7 | 20 | 16.1 | 17 | 13.6 | 23 | 18.3 | 27 | 21.4 | 17 | 13.7 | 18 | 14.4 | 165 | 16.5 |  |
| **Sex** |  |  |  |  |  |  |  |  |  |  |  |  |  |  |  |  |  |  |  |
| Male | 45 | 36.3 | 49 | 38.9 | 57 | 46.0 | 47 | 37.6 | 39 | 31.0 | 51 | 40.5 | 48 | 38.7 | 39 | 31.2 | 375 | 37.5 | 0.256 |
| Female | 79 | 63.7 | 77 | 61.1 | 67 | 54.0 | 78 | 62.4 | 87 | 69.1 | 75 | 59.5 | 76 | 61.3 | 86 | 68.8 | 625 | 62.5 |  |
| **Education** | |  |  |  |  |  |  |  |  |  |  |  |  |  |  |  |  |  |  |
| Below bachelor degree | 55 | 44.4 | 59 | 46.8 | 42 | 33.9 | 52 | 41.6 | 54 | 42.9 | 58 | 46.0 | 55 | 44.4 | 63 | 50.4 | 438 | 43.8 | 0.313 |
| Bachelor degree or above | 69 | 55.7 | 67 | 53.2 | 82 | 66.1 | 73 | 58.4 | 72 | 57.1 | 68 | 54.0 | 69 | 55.7 | 62 | 49.6 | 562 | 56.2 |  |
| **Household income** | | |  |  |  |  |  |  |  |  |  |  |  |  |  |  |  |  |  |
| Below HK$30,000 | 42 | 33.9 | 48 | 38.1 | 47 | 37.9 | 50 | 40.0 | 55 | 43.7 | 65 | 51.6 | 54 | 43.6 | 60 | 48.0 | 421 | 42.1 | 0.098 |
| HK$30,000+ | 82 | 66.1 | 78 | 61.9 | 77 | 62.1 | 75 | 60.0 | 71 | 56.4 | 61 | 48.4 | 70 | 56.5 | 65 | 52.0 | 579 | 57.9 |  |
| **Chronic conditions** | | |  |  |  |  |  |  |  |  |  |  |  |  |  |  |  |  |  |
| No | 107 | 86.3 | 111 | 88.1 | 114 | 91.9 | 109 | 87.2 | 104 | 82.5 | 113 | 89.7 | 102 | 82.3 | 111 | 88.8 | 871 | 87.1 | 0.271 |
| Yes | 17 | 13.7 | 15 | 11.9 | 10 | 8.1 | 16 | 12.8 | 22 | 17.5 | 13 | 10.3 | 22 | 17.7 | 14 | 11.2 | 129 | 12.9 |  |
| **Uptake of COVID-19 vaccine** | | | |  |  |  |  |  |  |  |  |  |  |  |  |  |  |  |  |
| No | 29 | 23.4 | 29 | 23.0 | 17 | 13.7 | 20 | 16.0 | 27 | 21.4 | 15 | 11.9 | 27 | 21.8 | 29 | 23.2 | 193 | 19.3 | 0.091 |
| Yes | 95 | 76.6 | 97 | 77.0 | 107 | 86.3 | 105 | 84.0 | 99 | 78.6 | 111 | 88.1 | 97 | 78.2 | 96 | 76.8 | 807 | 80.7 |  |
| **Perceived "likely/very likely" to be infected** | | | | | | |  |  |  |  |  |  |  |  |  |  |  |  |  |
| No | 84 | 67.7 | 79 | 62.7 | 77 | 62.1 | 80 | 64.0 | 86 | 68.3 | 83 | 65.9 | 84 | 67.7 | 89 | 71.2 | 662 | 66.2 | 0.803 |
| Yes | 40 | 32.3 | 47 | 37.3 | 47 | 37.9 | 45 | 36.0 | 40 | 31.8 | 43 | 34.1 | 40 | 32.3 | 36 | 28.8 | 338 | 33.8 |  |
| **Perceived "slightly severe/very severe" if get infected COVID-19** | | | | | | | | |  |  |  |  |  |  |  |  |  |  |  |
| No | 63 | 50.8 | 75 | 59.5 | 69 | 55.7 | 76 | 60.8 | 72 | 57.1 | 86 | 68.3 | 69 | 55.7 | 67 | 53.6 | 577 | 57.7 | 0.181 |
| Yes | 61 | 49.2 | 51 | 40.5 | 55 | 44.4 | 49 | 39.2 | 54 | 42.9 | 40 | 31.8 | 55 | 44.4 | 58 | 46.4 | 423 | 42.3 |  |
| **"Slightly/very" worry about being quarantined** | | | | | | |  |  |  |  |  |  |  |  |  |  |  |  |  |
| No | 45 | 36.3 | 44 | 34.9 | 41 | 33.1 | 40 | 32.0 | 39 | 31.0 | 41 | 32.5 | 43 | 34.7 | 41 | 32.8 | 334 | 33.4 | 0.991 |
| Yes | 79 | 63.7 | 82 | 65.1 | 83 | 66.9 | 85 | 68.0 | 87 | 69.1 | 85 | 67.5 | 81 | 65.3 | 84 | 67.2 | 666 | 66.6 |  |

Table S2. Vaccine acceptance among participants who did not received COVID-19 vaccine

|  | Refuse | | Accept | | Total | | P value |
| --- | --- | --- | --- | --- | --- | --- | --- |
|  | N | % | N | % | N | % |  |
| **Age** |  |  |  |  |  |  |  |
| 18-34 yrs | 86 | 69.4 | 40 | 58.0 | 126 | 65.3 | 0.232 |
| 35-49 yrs | 25 | 20.2 | 17 | 24.6 | 42 | 21.8 |  |
| 50+ yrs | 13 | 10.5 | 12 | 17.4 | 25 | 13.0 |  |
| **Sex** |  |  |  |  |  |  |  |
| Male | 39 | 31.5 | 22 | 31.9 | 61 | 31.6 | 0.951 |
| Female | 85 | 68.6 | 47 | 68.1 | 132 | 68.4 |  |
| **Education** | |  |  |  |  |  |  |
| Below bachelor degree | 54 | 43.6 | 43 | 62.3 | 97 | 50.3 | 0.012 |
| Bachelor degree or above | 70 | 56.5 | 26 | 37.7 | 96 | 49.7 |  |
| **Household income** | |  |  |  |  |  |  |
| Below HK$30,000 | 48 | 38.7 | 35 | 50.7 | 83 | 43.0 | 0.106 |
| HK$30,000+ | 76 | 61.3 | 34 | 49.3 | 110 | 57.0 |  |
| **Chronic conditions** | |  |  |  |  |  |  |
| No | 102 | 82.3 | 60 | 87.0 | 162 | 83.9 | 0.394 |
| Yes | 22 | 17.7 | 9 | 13.0 | 31 | 16.1 |  |
| **Perceived "likely/very likely" to be infected** | | | | |  |  |  |
| No | 83 | 66.9 | 39 | 56.5 | 122 | 63.2 | 0.150 |
| Yes | 41 | 33.1 | 30 | 43.5 | 71 | 36.8 |  |
| **Perceived "slightly severe/very severe" if get infected COVID-19** | | | | | | |  |
| No | 65 | 52.4 | 26 | 37.7 | 91 | 47.2 | 0.049 |
| Yes | 59 | 47.6 | 43 | 62.3 | 102 | 52.9 |  |
| **"Slightly/very" worry about being quarantined** | | | | |  |  |  |
| No | 39 | 31.5 | 17 | 24.6 | 56 | 29.0 | 0.317 |
| Yes | 85 | 68.6 | 52 | 75.4 | 137 | 71.0 |  |
| **Perceived "relatively high" safety of the vaccines** | | | | |  |  |  |
| No | 103 | 83.1 | 46 | 66.7 | 149 | 77.2 | 0.009 |
| Yes | 21 | 16.9 | 23 | 33.3 | 44 | 22.8 |  |
| **Perceived "relatively high" effectiveness of the vaccines** | | | | | |  |  |
| No | 101 | 81.5 | 37 | 53.6 | 138 | 71.5 | <0.001 |
| Yes | 23 | 18.6 | 32 | 46.4 | 55 | 28.5 |  |
| **Loss frame** | |  |  |  |  |  |  |
| No | 64 | 51.6 | 31 | 44.9 | 95 | 49.2 | 0.373 |
| Yes | 60 | 48.4 | 38 | 55.1 | 98 | 50.8 |  |
| **Physician consultation** | | |  |  |  |  |  |
| No | 72 | 58.1 | 28 | 40.6 | 100 | 51.8 | 0.020 |
| Yes | 52 | 41.9 | 41 | 59.4 | 93 | 48.2 |  |
| **Cash incentive** | |  |  |  |  |  |  |
| No | 63 | 50.8 | 37 | 53.6 | 100 | 51.8 | 0.707 |
| Yes | 61 | 49.2 | 32 | 46.4 | 93 | 48.2 |  |
| **Total** | 124 | 100.0 | 69 | 100.0 | 193 | 100.0 |  |

Table S3. Willingness to accept vaccine earlier than actually did among participants who received COVID-19 vaccine

|  | Refuse | | Accept | | Total | | P value |
| --- | --- | --- | --- | --- | --- | --- | --- |
|  | N | % | N | % | N | % |  |
| **Age** |  |  |  |  |  |  |  |
| 18-34 yrs | 131 | 60.9 | 301 | 50.8 | 432 | 53.5 | 0.021 |
| 35-49 yrs | 48 | 22.3 | 187 | 31.6 | 235 | 29.1 |  |
| 50+ yrs | 36 | 16.7 | 104 | 17.6 | 140 | 17.4 |  |
| **Sex** |  |  |  |  |  |  |  |
| Male | 77 | 35.8 | 237 | 40.0 | 314 | 38.9 | 0.277 |
| Female | 138 | 64.2 | 355 | 60.0 | 493 | 61.1 |  |
| **Education** | |  |  |  |  |  |  |
| Below bachelor degree | 85 | 39.5 | 256 | 43.2 | 341 | 42.3 | 0.346 |
| Bachelor degree or above | 130 | 60.5 | 336 | 56.8 | 466 | 57.7 |  |
| **Household income** | |  |  |  |  |  |  |
| Below HK$30,000 | 91 | 42.3 | 247 | 41.7 | 338 | 41.9 | 0.878 |
| HK$30,000+ | 124 | 57.7 | 345 | 58.3 | 469 | 58.1 |  |
| **Chronic conditions** | |  |  |  |  |  |  |
| No | 91 | 42.3 | 247 | 41.7 | 338 | 41.9 | 0.607 |
| Yes | 124 | 57.7 | 345 | 58.3 | 469 | 58.1 |  |
| **Perceived "likely/very likely" to be infected** | | | | |  |  |  |
| No | 143 | 66.5 | 397 | 67.1 | 540 | 66.9 | 0.883 |
| Yes | 72 | 33.5 | 195 | 32.9 | 267 | 33.1 |  |
| **Perceived "slightly severe/very severe" if get infected COVID-19** | | | | | | |  |
| No | 123 | 57.2 | 363 | 61.3 | 486 | 60.2 | 0.292 |
| Yes | 92 | 42.8 | 229 | 38.7 | 321 | 39.8 |  |
| **"Slightly/very" worry about being quarantined** | | | | |  |  |  |
| No | 84 | 39.1 | 194 | 32.8 | 278 | 34.5 | 0.096 |
| Yes | 131 | 60.9 | 398 | 67.2 | 529 | 65.6 |  |
| **Perceived "relatively high" safety of the vaccines** | | | | |  |  |  |
| No | 117 | 54.4 | 163 | 27.5 | 280 | 34.7 | <0.001 |
| Yes | 98 | 45.6 | 429 | 72.5 | 527 | 65.3 |  |
| **Perceived "relatively high" effectiveness of the vaccines** | | | | | |  |  |
| No | 124 | 57.7 | 185 | 31.3 | 309 | 38.3 | <0.001 |
| Yes | 91 | 42.3 | 407 | 68.8 | 498 | 61.7 |  |
| **Loss frame** | |  |  |  |  |  |  |
| No | 140 | 65.1 | 264 | 44.6 | 404 | 50.1 | <0.001 |
| Yes | 75 | 34.9 | 328 | 55.4 | 403 | 49.9 |  |
| **Physician consultation** | | |  |  |  |  |  |
| No | 125 | 58.1 | 273 | 46.1 | 398 | 49.3 | 0.003 |
| Yes | 90 | 41.9 | 319 | 53.9 | 409 | 50.7 |  |
| **Cash incentive** | |  |  |  |  |  |  |
| No | 100 | 46.5 | 302 | 51.0 | 402 | 49.8 | 0.258 |
| Yes | 115 | 53.5 | 290 | 49.0 | 405 | 50.2 |  |
| **Total** | 215 | 100.0 | 592 | 100.0 | 807 | 100.0 |  |

Table S4. Results of Model 3 (according to perceived infection risk of participants who did not receive vaccine)

|  | Not receive COVID-19 vaccine (Model 3, n=193) | |
| --- | --- | --- |
|  | AOR | 95%CI |
| Loss-framing (vs. gain-framing) | 2.17 | (0.80, 5.87) |
| Physician consultation (vs. no consultation) | 2.73* | (1.01, 7.38) |
| Cash incentive (vs. no cash) | 0.99 | (0.37, 2.64) |
| Perceived "likely/very likely" to be infected | 1.70 | (0.33, 8.86) |
|  |  |  |
| Loss-framing x higher perceived risk | 0.90 | (0.19, 4.39) |
| consult x higher perceived risk | 1.53 | (0.31, 7.51) |
| cash x higher perceived risk | 0.47 | (0.10, 2.19) |
|  |  |  |
| Age (18-34 yrs as reference) | |  |
| 35-49 yrs | 1.29 | (0.50, 3.32) |
| 50-64 yrs | 1.27 | (0.49, 3.27) |
| Female (vs. male) | 0.75 | (0.34, 1.68) |
| Chronic condition | 0.57 | (0.20, 1.63) |
| Bachelor degree or above | 0.46 | (0.19, 1.12) |
| HK$30,000+ monthly household income | 0.94 | (0.39, 2.25) |
| Perceived "slightly severe/very severe" if infected | 2.04 | (0.85, 4.90) |
| Perceived relatively high vaccine safety | 0.52 | (0.13, 2.16) |
| Perceived relatively high vaccine effectiveness | 9.05* | (2.35, 34.81) |
| "Slightly/very" concerned about being quarantined | 0.81 | (0.32, 2.06) |

Table S5. Results of Model 4 (according to perceived infection risk of participants who received vaccine)

|  | Receive COVID-19 vaccine (Model 4, n=807) | |
| --- | --- | --- |
|  | AOR | 95%CI |
| Loss-framing (vs. gain-framing) | 2.11** | (1.39, 3.21) |
| Physician consultation (vs. no consultation) | 2.03* | (1.34, 3.07) |
| Cash incentive (vs. no cash) | 0.98 | (0.65, 1.48) |
| Perceived "likely/very likely" to be infected | 1.18 | (0.58, 2.40) |
|  |  |  |
| Loss-framing x higher perceived risk | 1.32 | (0.62, 2.84) |
| consult x higher perceived risk | 1.14 | (0.54, 2.38) |
| cash x higher perceived risk | 0.81 | (0.39, 1.71) |
|  |  |  |
| Age (18-34 yrs as reference) |  |  |
| 35-49 yrs | 1.31 | (0.84, 2.04) |
| 50-64 yrs | 0.82 | (0.51, 1.32) |
| Female (vs. male) | 1.00 | (0.71, 1.42) |
| Chronic condition | 1.21 | (0.72, 2.02) |
| Bachelor degree or above | 0.89 | (0.59, 1.34) |
| HK$30,000+ monthly household income | 1.12 | (0.75, 1.67) |
| Perceived "slightly severe/very severe" if infected | 0.70 | (0.48, 1.02) |
| Perceived relatively high vaccine safety | 2.25** | (1.44, 3.52) |
| Perceived relatively high vaccine effectiveness | 1.96* | (1.24, 3.09) |
| "Slightly/very" concerned about being quarantined | 1.61* | (1.12, 2.32) |

Table S6. Results of Model 5 (according to perceived severity of participants who did not receive vaccine)

|  | Not receive COVID-19 vaccine (Model 5, n=193) | |
| --- | --- | --- |
|  | AOR | 95%CI |
| Loss-framing (vs. gain-framing) | 2.34 | (0.73, 7.53) |
| Physician consultation (vs. no consultation) | 2.44 | (0.74, 8.07) |
| Cash incentive (vs. no cash) | 0.99 | (0.29, 3.37) |
| Perceived "slightly severe/very severe" if infected | 2.14 | (0.45, 10.26) |
|  |  |  |
| Loss-framing x higher perceived risk | 0.77 | (0.17, 3.59) |
| consult x higher perceived risk | 1.59 | (0.34, 7.44) |
| cash x higher perceived risk | 0.61 | (0.13, 2.86) |
|  |  |  |
| Age (18-34 yrs as reference) | |  |
| 35-49 yrs | 1.28 | (0.49, 3.34) |
| 50-64 yrs | 1.22 | (0.47, 3.20) |
| Female (vs. male) | 0.79 | (0.35, 1.74) |
| Chronic condition | 0.57 | (0.20, 1.63) |
| Bachelor degree or above | 0.43 | (0.18, 1.07) |
| HK$30,000+ monthly household income | 0.92 | (0.38, 2.22) |
| Perceived "likely/very likely" to be infected | 1.49 | (0.62, 3.58) |
| Perceived relatively high vaccine safety | 0.49 | (0.12, 2.02) |
| Perceived relatively high vaccine effectiveness | 9.06* | (2.38, 34.46) |
| "Slightly/very" concerned about being quarantined | 0.83 | (0.33, 2.10) |

Table S7. Results of Model 6 (according to perceived severity of participants who received vaccine)

|  | Receive COVID-19 vaccine (Model 6, n=807) | |
| --- | --- | --- |
|  | AOR | 95%CI |
| Loss-framing (vs. gain-framing) | 1.72* | (1.10, 2.70) |
| Physician consultation (vs. no consultation) | 1.83* | (1.18, 2.86) |
| Cash incentive (vs. no cash) | 1.19 | (0.76, 1.86) |
| Perceived "slightly severe/very severe" if infected | 0.62 | (0.31, 1.22) |
|  |  |  |
| Loss-framing x higher perceived risk | 2.17* | (1.05, 4.47) |
| consult x higher perceived risk | 1.58 | (0.77, 3.24) |
| cash x higher perceived risk | 0.48* | (0.24, 0.97) |
|  |  |  |
| Age (18-34 yrs as reference) | | |
| 35-49 yrs | 1.29 | (0.82, 2.02) |
| 50-64 yrs | 0.81 | (0.50, 1.30) |
| Female (vs. male) | 1.00 | (0.70, 1.41) |
| Chronic condition | 1.23 | (0.74, 2.07) |
| Bachelor degree or above | 0.89 | (0.59, 1.34) |
| HK$30,000+ monthly household income | 1.11 | (0.75, 1.66) |
| Perceived "likely/very likely" to be infected | 1.23 | (0.84, 1.80) |
| Perceived relatively high vaccine safety | 2.27** | (1.45, 3.56) |
| Perceived relatively high vaccine effectiveness | 1.98* | (1.25, 3.14) |
| "Slightly/very" concerned about being quarantined | 1.67* | (1.16, 2.42) |

Table S8. Results of Model 7 (according to perceived vaccine safety of participants who did not receive vaccine)

|  | Not receive COVID-19 vaccine (Model 7, n=193) | |
| --- | --- | --- |
|  | AOR | 95%CI |
| Loss-framing (vs. gain-framing) | 2.58* | (1.01, 6.61) |
| Physician consultation (vs. no consultation) | 3.89* | (1.55, 9.77) |
| Cash incentive (vs. no cash) | 0.53 | (0.22, 1.31) |
| Perceived relatively high vaccine safety | 0.55 | (0.08, 4.09) |
|  |  |  |
| Loss-framing x higher perceived risk | 0.37 | (0.06, 2.24) |
| consult x higher perceived risk | 0.68 | (0.12, 3.82) |
| cash x higher perceived risk | 3.26 | (0.60, 17.82) |
|  |  |  |
| Age (18-34 yrs as reference) | | |
| 35-49 yrs | 1.36 | (0.52, 3.57) |
| 50-64 yrs | 1.27 | (0.48, 3.37) |
| Female (vs. male) | 0.76 | (0.35, 1.69) |
| Chronic condition | 0.58 | (0.20, 1.66) |
| Bachelor degree or above | 0.42 | (0.17, 1.08) |
| HK$30,000+ monthly household income | 0.90 | (0.36, 2.25) |
| Perceived "likely/very likely" to be infected | 1.47 | (0.61, 3.55) |
| Perceived "slightly severe/very severe" if infected | 1.87 | (0.78, 4.49) |
| Perceived relatively high vaccine effectiveness | 9.79* | (2.50, 38.29) |
| "Slightly/very" concerned about being quarantined | 0.89 | (0.35, 2.29) |

Table S9. Results of Model 8 (according to perceived vaccine safety of participants who received vaccine)

|  | Receive COVID-19 vaccine (Model 8, n=807) | |
| --- | --- | --- |
|  | AOR | 95%CI |
| Loss-framing (vs. gain-framing) | 1.73* | (1.00, 2.98) |
| Physician consultation (vs. no consultation) | 3.00** | (1.75, 5.15) |
| Cash incentive (vs. no cash) | 1.58 | (0.92, 2.73) |
| Perceived relatively high vaccine safety | 3.73** | (1.78, 7.81) |
|  |  |  |
| Loss-framing x higher perceived risk | 1.67 | (0.81, 3.42) |
| consult x higher perceived risk | 0.58 | (0.29, 1.17) |
| cash x higher perceived risk | 0.39* | (0.19, 0.80) |
|  |  |  |
| Age (18-34 yrs as reference) | | |
| 35-49 yrs | 1.32 | (0.85, 2.07) |
| 50-64 yrs | 0.83 | (0.52, 1.34) |
| Female (vs. male) | 1.01 | (0.72, 1.44) |
| Chronic condition | 1.28 | (0.76, 2.15) |
| Bachelor degree or above | 0.87 | (0.58, 1.31) |
| HK$30,000+ monthly household income | 1.11 | (0.74, 1.66) |
| Perceived "likely/very likely" to be infected | 1.24 | (0.85, 1.82) |
| Perceived "slightly severe/very severe" if infected | 0.70 | (0.48, 1.02) |
| Perceived relatively high vaccine effectiveness | 1.96* | (1.24, 3.12) |
| "Slightly/very" concerned about being quarantined | 1.63* | (1.13, 2.35) |

Table S10. Results of Model 9 (according to perceived vaccine effectiveness of participants who did not receive vaccine)

|  | Not receive COVID-19 vaccine (Model 9, n=193) | |
| --- | --- | --- |
|  | AOR | 95%CI |
| Loss-framing (vs. gain-framing) | 2.00 | (0.78, 5.14) |
| Physician consultation (vs. no consultation) | 3.40* | (1.34, 8.63) |
| Cash incentive (vs. no cash) | 0.45 | (0.17, 1.16) |
| Perceived relatively high vaccine effectiveness | 4.75 | (0.64, 35.41) |
|  |  |  |
| Loss-framing x higher perceived risk | 1.01 | (0.20, 5.11) |
| consult x higher perceived risk | 0.97 | (0.18, 5.27) |
| cash x higher perceived risk | 4.00 | (0.82, 19.57) |
|  |  |  |
| Age (18-34 yrs as reference) | | |
| 35-49 yrs | 1.24 | (0.47, 3.24) |
| 50-64 yrs | 1.15 | (0.43, 3.07) |
| Female (vs. male) | 0.78 | (0.35, 1.73) |
| Chronic condition | 0.58 | (0.20, 1.65) |
| Bachelor degree or above | 0.48 | (0.19, 1.21) |
| HK$30,000+ monthly household income | 0.83 | (0.34, 2.04) |
| Perceived "likely/very likely" to be infected | 1.38 | (0.58, 3.30) |
| Perceived "slightly severe/very severe" if infected | 1.92 | (0.81, 4.58) |
| Perceived relatively high vaccine safety | 0.50 | (0.11, 2.25) |
| "Slightly/very" concerned about being quarantined | 0.93 | (0.36, 2.39) |

Table S11. Results of Model 10 (according to perceived vaccine safety of participants who received vaccine)

|  | Receive COVID-19 vaccine (Model 10, n=807) | |
| --- | --- | --- |
|  | AOR | 95%CI |
| Loss-framing (vs. gain-framing) | 2.05* | (1.21, 3.47) |
| Physician consultation (vs. no consultation) | 1.63 | (0.97, 2.74) |
| Cash incentive (vs. no cash) | 1.19 | (0.70, 2.01) |
| Perceived relatively high vaccine effectiveness | 1.79 | (0.87, 3.69) |
|  |  |  |
| Loss-framing x higher perceived risk | 1.31 | (0.65, 2.67) |
| consult x higher perceived risk | 1.63 | (0.81, 3.28) |
| cash x higher perceived risk | 0.62 | (0.31, 1.24) |
|  |  |  |
| Age (18-34 yrs as reference) | | |
| 35-49 yrs | 1.35 | (0.86, 2.10) |
| 50-64 yrs | 0.84 | (0.52, 1.35) |
| Female (vs. male) | 1.02 | (0.72, 1.45) |
| Chronic condition | 1.24 | (0.74, 2.07) |
| Bachelor degree or above | 0.89 | (0.59, 1.34) |
| HK$30,000+ monthly household income | 1.11 | (0.75, 1.66) |
| Perceived "likely/very likely" to be infected | 1.25 | (0.86, 1.82) |
| Perceived "slightly severe/very severe" if infected | 0.70 | (0.48, 1.02) |
| Perceived relatively high vaccine safety | 2.26** | (1.44, 3.53) |
| "Slightly/very" concerned about being quarantined | 1.58* | (1.10, 2.27) |

Survey experiment questionnaire for COVID-19 vaccination

1. Your age in years：

- 17 or below
- 18 – 24
- 25 – 29
- 30 – 34
- 35 – 39
- 40 – 44
- 45 – 49
- 50 – 54
- 55 – 59
- 60 – 64
- 65 or above

1. Your gender**:**

- Male
- Female

1. How likely do you think you may be infected with COVID-19?

- Very unlikely
- Unlikely
- Likely
- Very likely

1. If you are unfortunately infected with COVID- 19, how serious do you think your condition could be?

- Completely not serious
- Not so serious
- A little serious
- Very serious

1. How do you concern about yourself being quarantined at the quarantine camp due to contact with COVID-19 patients or living in the same building with COVID-19 patients?

- Not concerned at all
- Not so concerned
- Somewhat concerned
- Very concerned

1. How do you feel about the safety of the COVID-19 vaccine?

|  | 0 | | 1 | | 2 | | 3 | | 4 | | 5 | | 6 | | 7 | | 8 | | 9 | | 10 | |  |
| --- | --- | --- | --- | --- | --- | --- | --- | --- | --- | --- | --- | --- | --- | --- | --- | --- | --- | --- | --- | --- | --- | --- | --- |
|  | |  | |  | |  | |  | |  | |  | |  | |  | |  | |  | |  | |
| Very unsafe | | | |  | |  | |  | |  | |  | |  | |  | |  | | Very safe | | | |

1. How do you feel about the effectiveness of the COVID-19 vaccine?

|  | 0 | | 1 | | 2 | | 3 | | 4 | | 5 | | 6 | | 7 | | 8 | | 9 | | 10 | |  |
| --- | --- | --- | --- | --- | --- | --- | --- | --- | --- | --- | --- | --- | --- | --- | --- | --- | --- | --- | --- | --- | --- | --- | --- |
|  | |  | |  | |  | |  | |  | |  | |  | |  | |  | |  | |  | |
| Very ineffective | | | |  | |  | |  | |  | |  | |  | |  | |  | | Very effective | | | |

1. Have you received at least one dose of COVID-19 vaccine?

- No
- Yes

1. How many doses of COVID-19 vaccine have you received?

- One
- Two

1. Survey experiment questions

**(Each respondent randomized into one of arm 1-8 of the survey experiment)**

- 1. Survey experiment – Arm 1

1. The probability of COVID-19 infection of individual who receive the vaccine are 50%-95% lower (i.e. 1/2 – 1/20) than those who did not get the vaccine.

**Would you choose to accept the vaccine (earlier) out of consideration of these elements?**

- No
- Yes
  1. Survey experiment – Arm 2

1. The probability of COVID-19 infection of individual who receive the vaccine are 50%-95% lower (i.e. 1/2 – 1/20) than those who did not get the vaccine;
2. Assume that the government provides one subsidized consultation by a doctor of your choice prior to vaccination to assess your suitability of vaccination, and the doctor tells you that you are suitable.

**Would you choose to accept the vaccine (earlier) out of consideration of these elements?**

- No
- Yes
  1. Survey experiment – Arm 3

1. The probability of COVID-19 infection of individual who receive the vaccine are 50%-95% lower (i.e. 1/2 – 1/20) than those who did not get the vaccine;
2. Assume that the vaccinated individuals can receive HK$500 in cash.

**Would you choose to accept the vaccine (earlier) out of consideration of these elements?**

- No
- Yes
  1. Survey experiment – Arm 4

1. The probability of COVID-19 infection of individual who receive the vaccine are 50%-95% lower (i.e. 1/2 – 1/20) than those who did not get the vaccine;
2. Assume that the vaccinated individuals can receive HK$500 in cash;
3. Assume that the government provides one subsidized consultation by a doctor of your choice prior to vaccination to assess your suitability of vaccination, and the doctor tells you that you are suitable.

**Would you choose to accept the vaccine (earlier) out of consideration of these elements?**

- No
- Yes
  1. Survey experiment – Arm 5

1. The probability of COVID-19 infection of individual who did not receive the vaccine are 2-20 times higher than those who received the vaccine

**Would you choose to accept the vaccine (earlier) out of consideration of these elements?**

- No
- Yes
  1. Survey experiment – Arm 6

1. The probability of COVID-19 infection of individual who did not receive the vaccine are 2-20 times higher than those who received the vaccine
2. Assume that the government provides one subsidized consultation by a doctor of your choice prior to vaccination to assess your suitability of vaccination, and the doctor tells you that you are suitable

**Would you choose to accept the vaccine (earlier) out of consideration of these elements?**

- No
- Yes
  1. Survey experiment – Arm 7

1. The probability of COVID-19 infection of individual who did not receive the vaccine are 2-20 times higher than those who received the vaccine
2. Assume that the vaccinated individuals can receive HK$500 in cash

**Would you choose to accept the vaccine (earlier) out of consideration of these elements?**

- No
- Yes
  1. Survey experiment – Arm 8

1. The probability of COVID-19 infection of individual who did not receive the vaccine are 2-20 times higher than those who received the vaccine
2. Assume that the vaccinated individuals can receive HK$500 in cash
3. Assume that the government provides one subsidized consultation by a doctor of your choice prior to vaccination to assess your suitability of vaccination, and the doctor tells you that you are suitable

**Would you choose to accept the vaccine (earlier) out of consideration of these elements?**

- No
- Yes

1. What is the highest level of education you have attained?

- Primary school or below
- Secondary school
- Diploma/certificate course
- Sub-degree course
- Degree course or above

1. What is the range of your monthly household income?

- Below HK$10,000
- HK$10,000 – HK$19,999
- HK$20,000 – HK$29,999
- HK$30,000 – HK$39,999
- HK$40,000 – HK$49,999
- HK$50,000 or above

1. Have you ever been diagnosed to have any chronic conditions?

- No
- Yes
